# Entrepreneurship challenges: the case of Jordanian start-ups

**DOI:** 10.1186/s13731-023-00286-z

**Published:** 2023-04-05

**Authors:** Mohammad Alawamleh, Yara Hanna Francis, Kamal Jamal Alawamleh

**Affiliations:** 1 Business Administration Department, American University of Madaba, Madaba, Jordan; 2 Faculty of Law, University of Petra, Amman, Jordan

**Keywords:** Entrepreneurs, Start-ups, Challenges, Jordan

## Abstract

This study investigates challenges facing entrepreneurs in Jordan, identifies some of problems specific to SMEs, and offers some solutions to help these companies improve their conditions. This study collected primary data through interviews with entrepreneurs in four start-up companies specialized in diverse fields, including information technology, consulting, training, and e-marketing. This study found many obstacles facing entrepreneurs in Jordan, the most important of which are financial and logistical support, the challenge of distributing work, owning operating experiences, obtaining information, commercial relations, and networking. The results of this study showed that women face greater challenges in relation to financing and investment opportunities. Finally, this study proposes some solutions which expected to be applied to improve the business environment, the most important of which is that the Jordanian Ministry of Digital Economy and Entrepreneurship should activate pilot projects and stimulate investment in them, facilitating the development and deployment of technical knowledge in private sector development.

## Introduction

Entrepreneurship has been an increasingly important subject of academic studies since the 1980s, due to the great socio-economic importance of the phenomenon in socio-economic development under neoliberal monetarist policies. The modern economy is significantly based on entrepreneurs’ ability to create new ideas and product that generate employment, resulting in the expansion, growth, and development of local and national economies, and global economic development in general (Hessels & Naudé, 2019). Moreover, while entrepreneurship provides benefits in terms of social and economic growth, it also offers benefits in terms of individual fulfilment (Cooney, 2012). Entrepreneurship is all about identifying various gaps presented within the market, developing new ideas and translating these ideas into products and actions to fill in these gaps (Chen et al., 2015).

Economies throughout the Middle East and North Africa (MENA) vary considerably, from countries with relatively high-value human resource potential but poor natural resources, such as Jordan and Tunisia, to resource rich countries with relatively limited labor opportunities for nationals, such as the oil and gas exporting economies of the Gulf Cooperation Council (GCC). However, all countries in the region share common factors of macro-economic instability, including high private sector unemployment, overemployment in the public sector, high sensitivity to fluctuations in oil and gas prices, and vulnerability due to conflicts in neighboring countries. The whole region requires a thorough and challenging socio-economic transformation, which necessitates increased focus on private sector development, and this requires producing building entrepreneurs who can convert ideas into successful business ventures, and managers who can streamline business processes, formulate and implement effective operations strategies, and lead to successful competitive advantages. It is essential to promote entrepreneurship to facilitate private sector growth and development, to facilitate economic diversification and long-term financial stability and socio-economic development.

This study explores some latent challenges confronting tentative entrepreneurship in Jordan, specifically the barriers facing Jordanian entrepreneurs seeking to enter the business world with new ventures (i.e., start-ups). Popular interest in start-ups has increased in recent years, especially among young people who are more alert to the possibilities of modern technological solutions to achieve their aspirations to establish personal projects. This has been commensurate with local and global economic deterioration and uncertainty, including large-scale unemployment and a lack of job opportunities.

Young people realize that the path is not easy and that it is full of difficulties when it comes to their personal projects. SMEs success play an important role of the success of the country’s economy. Mwatsika (2021) noted that entrepreneurship literature posits multiple perceptions to guide entrepreneurship development initiatives, but the dominant understanding of entrepreneurship is concerned with starting and managing one’s own business and being self-employed; this reflects the common understanding of entrepreneurship which guides entrepreneurship development at the national level.

National governments commonly fund projects seeking to develop the private sector to promote long-term economic growth, increasing investment opportunities, and pushing the wheel forward, providing employment that is essential for socio-economic development and healthy communities and societies. The economy of Jordan is considered a constricted and difficult competitive environment, with many obstacles facing the success of entrepreneurship. Entrepreneurs demand that the government constantly find solutions related to start-ups and SMEs. The challenges vary between financing, investment, human resources, the ability to attract customers and network with important sectors. This study identifies and explores such problems through data and information collection methods to find appropriate solutions to these challenges.

## Literature review

### Entrepreneurship

Entrepreneurship is considered one of the main business branches which have witnessed huge development over the last few decades. Success in today’s business environment relies, to a large extent, on the entrepreneurs who can convert ideas into successful businesses. Such success demand focus on entrepreneurship, as well as innovation, product development, applying good management practices, and meeting customer expectations and market requirements (Hessels & Naudé, 2019). Entrepreneurs have become strategically vital for modern organizations’ success, but their survival and sustaining their long-term existence in increasingly hostile and complex business environments is highly challenging, prompting extensive academic analyses of entrepreneurship from various angles. The science of entrepreneurship is usually concerned with the early stages of companies. Start-up companies are companies which have been in the market for less than 3 years, or that is still looking for fund or investment opportunities in its initial stages (Acosta, et al., 2018; Al-Dajani & Marlow, 2016).

Mehtap (2014) defined entrepreneurship as the science that is concerned with studying the development of companies’ work, starting from the first foundational stages to advanced businesses. Al-Dajani and Marlow (2016) classified entrepreneurship into that aiming purely at short-term economic profit; and societal entrepreneurship, which attempts to develop services and products that have greater long-term value for broader societal stakeholders and the environment. Societal entrepreneurs seek to improve the quality of people’s lives over the long term while also seeking economic viability in the short and medium terms.

Many researchers tried to dig deeper into the term entrepreneurship, as some of them specialized in studying the qualities and characteristics of a successful entrepreneur. Many researchers have concluded that there has been a development in the concept of entrepreneurship itself during recent years (Boldureanu et al., 2020). Many researchers believe that the term entrepreneurship has been closely related to the term “business administration”, but the science of entrepreneurship has developed very dramatically as a distinct discipline over the past few years. Several researchers have made different proposals regarding the origins of the science of entrepreneurship (Steininger, 2019), with some tracing it back as far as the early 1980s, which provided a suitable environment for managing business related to SMEs (Mehtap, 2014). Many countries have implemented the same strategy that the US followed by adopting business incubators to help start-ups and SMEs in a way that ensures the provision of support and networking with major companies and other various famous services (Weldali, 2020).

Many universities worldwide have included entrepreneurship specializations in their academic service delivery, either as distinct programs or as courses within business studies. This reflects the expansion of entrepreneurship specialization, and the urgent need of individuals and companies to understand and leverage it in an increasingly competitive global economy. Many studies have mentioned that the term entrepreneurship is closely related to the term “creativity”, in which regard entrepreneurship is common associated with technological and technical developments. Since the late twentieth century the ICT revolution, including the popularization of the Internet and the development of e-commerce, have greatly accelerated the trajectory of start-up companies offering new or niche products or services, especially those specifically related to technology itself and online services (Streeter et al., 2002).

There are many examples of companies that started as pioneering start-ups which have subsequently become among the most important and largest companies in the world, including Amazon. The pace at which entrepreneurship firms usually go varies, as some start-ups move and grow quickly, while others need more time to develop and achieve their goals. The emergence of the Internet has dramatically increased the potential scope of entrepreneurship (Elia et al., 2020; Fang et al., 2021). Many companies have started turning to the electronic platform as one of the opportunities through which they can increase their sales and related investment opportunities (Berman, 2012).

Companies have not only relied on the Internet to market products, but have mainly relied on it to work and search for solutions that help them sustain their business, and find new opportunities and innovative ways to accomplish tasks (Kraemer & Dedrick, 2002). However, while some of the most spectacular start-up success stories in history have been enabled by the modern e-commerce paradigm and Internet economy, there have been innumerable failures at the same time, due to various latent market and financial obstacles, and barriers to entrepreneurship itself. Concerning the latter, Morales et al. (2022) identified culture as the main obstacle, particularly the absence of an entrepreneurial culture in collectivist societies, which hampers and diminishes the creation of new business initiatives. Furthermore, an absence of family support, institutions communicating their efforts to promote entrepreneurship, sources of financing, “incubators”, and the absence of an entrepreneurial ecosystem are all considered as symptoms of cultural barriers to entrepreneurship.

### Entrepreneurship and start-up companies in Jordan

Previous studies showed that the business sector has developed significantly in Jordan during the past years. The Jordanian government has taken many incentive measures with the aim of trying to increase the demand for entrepreneurship and emerging projects by strengthening the system for supporting companies (Kreitmeyr, 2019). The business environment and Jordanian companies are specifically affected by several measures, the most important of which are the general economic situation in the country, the development of infrastructure, and general technical development (Alawamleh et al., 2019). Based on a survey of more than 230 entrepreneurs, it was found that most of them have a good level of education, in addition to having a good experience compared to others (Weldali, 2020). Another survey of start-ups in Jordan found that 62% of the companies have more than 10 years of experience in the business field (Mehtap, 2014). Entrepreneurs in Jordan try to work in appropriate groups as part of their business processes and to reach the skills that will enable them to continue to develop, and most emerging entrepreneurship companies in Jordan aspire to increase their sales despite the many adverse economic fundamentals that surround them (Caputo et al., 2016).

Start-ups and innovative SMEs tend to be considered as agents of change in economy, since they implement new products and services as well as more effective practices. They help to stimulate private sector growth and provide employment opportunities, which is essential to develop communities and spur economic development. In the context of Jordan, this is particularly important, because the unemployment rate has been increasing continuously in recent years, exacerbated by Jordan’s massive influx of refugees from neighbouring conflicts over recent decades. Government support and frameworks are necessary to incubate start-ups, to facilitate their long-term sustainability (Karani & Mshenga, 2021). Nowadays, many incubators and funding entities have established to support youth entrepreneurship and boost youth employment in Jordan. Current business tendencies and the Jordanian government’s 2019/2020 Priorities Plan encourage entrepreneurship as an important economic driver. Moreover, 98% of newly registered companies in the country are SMEs and start-ups, which generate more than 50% of private sector GDP, and 60% of new employment opportunities (UNICEF Jordan, 2019).

Many government agencies, such as the Ministry of Digital Economy and Entrepreneurship, have tried to encourage and support the entrepreneurial sector in Jordan by reducing the fees for establishing and registering companies. The Jordanian government has issued extensive legislation to support businesses, in cooperation with the Ministry of Digital Economy and Entrepreneurship, Chambers of Industry, Chambers of Commerce, and others with the aim of supporting companies’ infrastructure and finding new incentive solutions (Government of the Hashemite Kingdom of Jordan, 2021). Analyses of the Jordanian private sector have identified several basic categories of public–private formations supporting entrepreneurial companies, the most important of which are business incubators, business accelerators, and institutions that support technology companies (Weldali, 2020). One of the examples of financial supporters’ companies in entrepreneurship field in Jordan is Oasis500, a funding company which focuses its investment in start-up companies who are in their initial stages. In addition, the Queen Rania Center for Entrepreneurship is an incubator aiming to develop the technological infrastructure in Jordan. One of the goals of Queen Rania Center for Entrepreneurship is to enhance the networking between several start-ups.

Recently, telecommunications companies have exploited the increasing demand on entrepreneurship from individuals, companies and government. Thus, several companies have launched platforms that support the ecosystem of entrepreneurship, innovation and start-up companies. Zain Telecommunication Company launched Zain Innovation Campus (ZINC), and the Orange company launched the Business Incubation Growth (BIG), while Shamal Start is a famous leading business accelerator in Jordan (Jordan Times, 2018). Shamal Start is popular with the digital fabrication, including different manufacturing processes including robotics, assembly, and 3D printing.

### Entrepreneurship challenges in Jordan

There are many challenges that face the Jordanian start-ups and entrepreneurs. This section focuses on identifying the main challenges which was reported by companies and individuals in general (Al-Hawary & Al-Saysneh, 2020). One of the most fundamental challenges to entrepreneurship in Jordan is the massive taxation rates levied on companies and individuals in national fiscal policy. A recent report by the World Bank (2019) indicated that many start-ups can face tax rates. Based on a survey of 200 Jordanian entrepreneurs, they indicated that start-ups face numerous barriers to establishing their business operations in Jordan, ranging from inadequate policies and financial instruments to limited access to talent. The Global Entrepreneurship Index (2018) underlines that Jordan’s entrepreneurship ecosystem lags in high growth, risk capital, risk acceptance, networking, and human capital indicators.

Analysis of the literature indicates that there are many challenges facing entrepreneurs’ before and during the operation of their start-ups, based on which the following research questions were devised for the current inquiry:

- What are the difficulties that entrepreneurs face before starting their businesses?
- What are the challenges facing entrepreneurs during running their businesses?
- What are the key solutions to these challenges?

## Methodology

The study methodology aims to clarify the methods that will be followed during the study to reach the results. Study methodologies differ from one study to another, but many studies are similar in methodologies (Alawamleh, 2012). The methodology part consists of many sections, the most important of which are the type of study, the special sources in the study, the method of data and information collection, and an overview of the data analysis methods.

### Type of the study

There are two main types of studies, the first type is quantitative studies, and the second type is qualitative studies. Quantitative studies are defined as studies that rely more on numbers or measurements. Qualitative studies are defined as those studies that are expressed through descriptive speech (Bani Ismail, 2012). Each type of study has its own way of collecting data and information. Quantitative studies usually use questionnaires mainly and depend on converting questions into numbers to facilitate the calculation of their own measures.

This study relied more on qualitative investigation, exploring the subjective perceptions of Jordanian start-up entrepreneurs based on their experiences. Qualitative studies are based on interviews with individuals through open discussions and focused meetings (Alawamleh et al., 2020). Qualitative approaches probe such issues in-depth, and allow for the emergence of new and unforeseen aspects of inquiry, but their import is of limited generalizability due to the relatively low number of participants.

### Study sources

Researchers are usually interested in studying and knowing the sources of information in any research or study they conduct. There are two main sources for obtaining data in studies, primary and secondary sources. Primary sources are defined as the sources in which the researcher is highly relied upon through the information he collects through questionnaires, surveys, personal interviews, group interviews, dialogues, discussions and focus groups. Many studies rely on primary sources as an essential part of the study sources (Alawamleh et al., 2018). The second type is secondary sources, which are defined as the references and tools that the researcher uses to know the findings of the latest studies related to the same topic of study (Alawamleh et al., 2019). There are many examples of secondary sources that are usually used in the literature, the most important of which are scientific journals, reliable sources, the Internet, master’s theses and academic references (Alawamleh et al., 2020).

This study relies mainly on the primary sources collected by the researcher, and secondary sources by framing the research questions and interpreting the results of primary data in relation to existing literature, reviewing and summarizing the major findings on challenges to entrepreneurship in Jordan.

### Data collection instrument

This qualitative research depends on face-to-face interviews with the targeted sample under investigation, entrepreneurs in Jordanian start-ups. Interviews are the standard technique for collecting original data related to an in-depth research topic (Modell, 2007). Semi-structured interviews with representatives from four different start-up businesses in Jordan were conducted to collect the required information. This method was used, because it joined structured and unstructured interviews together and used open-ended questions, which allowed the interviewer and the interviewee to openly discuss the topic (Cooper & Schindler, 2014; Rogers et al., 2011).

Faulkner (2000) and Crabtree and Miller (1999) mentioned that interview goals related to the research topic should be established to be able to design the interview questions. The interview goals of this study included determining the nature of the businesses and their years of operation, which was addressed in Part 1 (with four questions). To understand the challenges faced by start-up companies in Jordan, questions were asked in Part 2 focusing on general challenges, and particular comparison of the situation of start-ups prior to and since the advent of the COVID-19 pandemic in 2020. Part 2 also asked interviewees to determine the best and worst years from the initiation of the start-up.

Part 3 of the study questionnaire asked about the Jordanian government’s role in enhancing the development of start-ups, and encouraging entrepreneurs, with four main questions. The first question asked respondents if they thought the Jordanian government had undertaken positive steps towards helping start-ups and economy. The second question asked their suggestions about what the government should do in the future to improve, enhance, and attract more start-ups. The last question elicited proposed solutions to improve the entrepreneurship ecosystem and milieu for start-ups in Jordan.

In addition to the open-ended questions, probing questions were used as needed, to allow the respondents to further explain and build on their responses, and for the researchers (who also conducted the interviews) to probe areas of emergent interest (Saunders et al., 2009). This method guarantees obtaining a full and in-depth understanding and detailed information about the interviewee’s perspectives. Interviewees were given the needed time to convey their openings and to respond to the questions without interruption, thus applying the rules of interviewing which govern most human interactions, no pre-judgments or assumptions (Crabtree & Miller, 1999; Modell, 2007).

### Target population and sampling

The target population of this study is entrepreneurs of start-up companies in Jordan. The study sample ought to be representative of the target population even in qualitative studies, where generalization is not a primary concern, and random sampling of participants meeting the criteria of the target population is preferred for this purpose. The sample in this study included entrepreneurs of both genders who have already started their own personal projects and who qualify as start-up owners. Start-up companies are companies that range in age from a few months to approximately 3 years. Start-ups need all kinds of support in this period to be able to continue their business. Examples of the support that start-ups try to obtain are logistical support, technical support, and financial support through investment opportunities, networking, and others.

This study attempts to reach the answers you are looking for and answer the main study questions by interviewing four different experts who own start-up companies to try to identify the nature of the challenges they face and the impact of the COVID-19 pandemic on their businesses.

### Interview ethics

To ensure that the interview was ethically conducted, the aims and objectives of the research were explained to the interviewees prior to starting the interview. In addition, the questions were reviewed and revised to ensure that they do not contain any harmful words (Cooper & Schindler, 2014), and the consent form included the researcher’s contact information and the respondents were made aware that they can withdraw their participation (Rogers et al., 2011).

Furthermore, participants themselves choose suitable places to meet, convenient to themselves and safe. In addition, each participant’s details were kept anonymous. It was clear for each participant from the consent form that the interview will be recorded and notes would be taken during the meeting; all respondents have confirmed their acceptance by signing the informed consent form.

## Analysis and results

The analysis part aims to discuss the results of the interviews that were conducted with the sample under study. This study focuses mainly on the issue of challenges faced by Jordanian women in the field of entrepreneurship and innovative projects. Many previous studies have attempted to identify the most important difficulties and challenges faced by women entrepreneurs in different environments, and some have identified particular issues faced by women in MENA countries concerning SME entrepreneurial projects (Abadi et al., 2022).

### Interviews analysis

This section presents and the most prominent opinions that arose from the thematic analysis of participants’ narrations in response to the 14 research questions derived from the literature review, along with follow-up questions that arose during the interviews themselves, as explained previously. The four interviewees, who are entrepreneurs working in start-up companies in Jordan, were asked about the main challenges they face during their work in the entrepreneurship sector in Jordan. Participants were from four different companies, anonymized as Company A (CA), Company B (CB), Company C (CC), and Company D (CD).

#### Company A (CA)

The first interview took place with one of the start-up companies working in the IT sector in Jordan. CA was a smaller start-up company founded 3 years ago, almost a year before the start of the COVID-19 pandemic.

The work in CA is divided between different genders, and CA is trying hard to sustain its business in an easy and fast way. A female co-founder of CA was asked about the difficulties that the company faces during its work, and she cited many issues related to substantial financial difficulties, especially in its early stages, and organizing its expansion plan in markets locally and internationally. Moreover, the CA team is trying hard to network with other companies to expand.

According to the co-founder of the entrepreneurship company, despite the great challenges before the COVID-19 pandemic, the situation was easier due to the financial decimation of Covid lockdowns on many economic sectors, which has resulted in many losses, closures, and declining profits. When asked about the best time in the business since its establishment, the co-founder stated that the pre-COVID era was the optimum time, especially in terms of marketing.

The third section of the questionnaire focused on the role of the Jordanian government in supporting the entrepreneurship sector. The participant mentioned that Jordan, through the Ministry of Digital Economy and Entrepreneurship, has taken many measures to support start-ups, but these measures are not enough.

#### Company B (CB)

The second interview took place with a training and consulting start-up company in Jordan. CB is considered particularly representative of current start-ups, as it was established during the last year. CB is considered small in size, with only four employees, distributed within several specializations and at different ages. The second part of the study attempted to identify the most important challenges CB faces, and a female entrepreneur identified many difficulties, including bureaucratic barriers in terms of licenses, approvals, and choosing the type of company for the purposes of registration.

One of the most prominent difficulties that CB faced is the financial issues related to the capital, as companies in Jordan commonly suffer greatly from poor access capital during the early stages. In addition, CB faced difficulties related to market studies, as it was not able to understand customers effectively during its inception period.

When talking about the most prominent difficulties that CB faced during the COVID-19 time, companies in general have gone through harsh periods due to closures and as a result of the great fear that occurred among customers.

The results of the interview indicated that there are many challenges facing female entrepreneurs in Jordan. One of the key challenges is having operational experience, as women entrepreneurs in general suffer from poor technical experience to manage projects, which leads them to resort to help from other people to sustain the business.

Based on the interview, it became clear that one of the most important challenges is the programs for developing entrepreneurship and sustainability and expanding the project in the future. In addition, they considered that obtaining information about the entrepreneurship support may as difficult and a great challenge, no less important than obtaining financial support.

#### Company C (CC)

The third interview took place with one of the companies specialized in market research studies. CC is considered small in size, and is trying to denounce the provision of market studies services for SMEs. Based on the interview with the female entrepreneur, the sector of entrepreneurship in Jordan suffers from many challenges.

The interviewee indicated that the number of female entrepreneurs working in the field of entrepreneurship constitutes less than a quarter of the total number of entrepreneurs. She also explained that there is often a partner for women working in the field.

The entrepreneur considered that one of the most important challenges facing women in the entrepreneurship sector in Jordan is financial support, as few entrepreneurial companies are incubated or have their work accelerated. Another important challenge is the lack of sufficient confidence, as many projects run by women fail during the first years of their work due to fear of failure or legal accountability, which pushes women to enter the courts in the event that they incur any financial sums in the event of the failure of the project, which is considered one of the most important challenges among major societal issues.

#### Company D (CD)

The fourth interview took place with a social media marketing company. CD is medium-sized, and focuses on providing marketing and consulting services on social media. CD activates advertising campaigns by focusing on developing work proposals and future marketing plans. CD has signed many special contracts with companies which enabled it to grow and develop, especially during a period that came with a significant increase in reliance on the establishment of e-marketing. The results of the interview showed that that there are many obstacles facing entrepreneurs in Jordan, especially females in Jordan. The most prominent of these obstacles are poor financial support, and attracting investors to invest in start-ups. The findings also showed that the challenges facing women entrepreneurs in Jordan include issues of appropriate networking with companies, as networking is could be make a difference for start-ups, especially to find potential customers.

### Discussion

After completing the analysis of the interviews with the study sample, this section attempts to summarize the most prominent findings of the study. The study showed that there are many difficulties that business companies in Jordan suffer from, most notably in the issue of financing their projects, as mentioned in previous studies. According to Marlow and Patton (2005), entrepreneurs face fundamental barriers due to financial weakness, sourcing funding and capital to support real investment opportunities.

Young Jordanians suffer from uncertainty and a lack of belief in themselves, which results in a pessimistic, insecure generation, who cannot innovate or lead the country to a better economic status. Ego also plays a role, because most Jordanians will not accept an opportunity that shows them starting small, as everyone wants to start at the top; this is not possible without the presence of connections, another major issue. Consequently, the confluence of ego culture and having the right connections results in a generation of unskilled professionals, working for the sole purpose of passing time and being paid. The unmotivated person with connections is given the job over someone who might be passionate about the work, who could have done better: a waste of a potential innovator and a wasted investment opportunity. In addition, the national economy will never advance if wasta keep causing opportunities to be missed.

One of the most difficult difficulties related to the work of business companies is the lack of practical experience in the business sector, and the study indicated that more than 60% of companies fail as a result of poor experience and skills. One of the most prominent challenges faced by entrepreneurs is the high project costs and rents, which leads to project suspension. Logistics services constitute a major obstacle for entrepreneurs, because understanding the market is essential in this field.

Entrepreneurs need programs that help develop their capabilities to reach high levels of work proficiency, and communication and knowledge of public relations is one of the biggest things that are required. The findings indicated that a large number of women start their businesses from home, which is what they already did before going to the bureaucratic obstructions of licensing, as the nature of their work did not typically require the presence of huge office equipment or decoration and meeting rooms at the beginning. They tended to use remote working options without the need for the many complications of formal business infrastructure and requirements, particularly during the COVID-19 lockdown era.

Familiarity with the regulations, laws and techniques of government institutions and departments is one of the most important obstacles facing entrepreneurs in Jordan, as the study indicated that the legal environment is one of the challenges for entrepreneurial projects, as it sometimes requires going through many long procedures that entrepreneurs are ignorant of. The companies’ knowledge of these matters is essential, which helps to sustain business, achieve gains and legal protection. This relates to the general stifling and unnecessarily complex nature of bureaucracy and state regulation across MENA countries, which is associated with low economic productivity and high levels of corruption, as noted by the Organisation for Economic Co-operation and Development (2010).

Entrepreneurship represents new, unfamiliar ideas, and is one of the ways of representing innovation, through the application of human capital’s innovative ideas to new businesses. However, Jordanian culture is generally slow to accept change, or new and unfamiliar ideas, due to a high level of uncertainty avoidance (Hofstede Insights, 2023). Consequently, it is necessary to change the way the younger generation perceives knowledge, education, opportunities, power distance, and entrepreneurship itself to promote the macroeconomic goals of entrepreneurship development in Jordan.

## Conclusion

This study attempted to answer many questions related to identification and exploration of barriers to entrepreneurship in Jordan. This study summarized the key difficulties faced by this sector, and tried to clarify the details of the work and what are the expected solutions to improve the current situation. The data gathered from qualitative interviews with entrepreneurs in four start-up SMEs in Jordan concluded that there are many challenges facing entrepreneurs in the country, the most prominent of which challenges are having operational experience and starting projects. Young entrepreneurs face many problems, including in relation to the skills and experience required in the market. The challenge of expanding the business is considered one of the challenges facing any entrepreneur, which stands in the way of expanding and obtaining more returns for the business.

The expansion stage is one of the most important stages in the plan of any business project. Interviewees indicated that access to financial services and investment opportunities is one of the biggest challenges faced by Jordanian entrepreneurs in particular. It is difficult for an entrepreneur to build relationships and networks on a large scale without the help of business incubators and accelerators. Finally, the interviewees indicated that a lack of techniques and knowledge of the legal environment, labor laws, and intellectual property rights pose obstacles facing entrepreneurs in Jordan.

The field of entrepreneurship in Jordan is highly competitive, as there are many ideas to initiate as well as many opportunities and centers that support entrepreneurial ideas and projects. This pillar is one of the most important dimensions of developing innovative human capital, because if it is encouraged, implemented, and applied effectively, this will provide entrepreneurial opportunities per se, and will enable the next generation to cover the skills gap, directly promoting national economic development and global recognition of Jordan’s human capital.

## Recommendations

The key recommendations of this study include the following:

1. It is necessary to activate the entrepreneurship sector with legislation and regulation encouraging start-up projects and facilitating business development (e.g., simplified and less bureaucratic processes, and tax advantages for new local companies).
2. Business accelerators and business incubators should invest in and spend more on pilot projects, which is essential for future socio-economic development in the Jordanian context.
3. Entrepreneurs should develop their technical skills and skills related to the legal environment, which might be enhanced by public–private partnerships and arrangements with local universities, and they must address the necessity of personnel having operational experience.
4. More networking and building large sustainable partnerships that help open up internal and external horizons is necessary (e.g., facilitated by government ministries holding conferences and social events for start-ups).

## Data Availability

Not applicable.

## Abbreviations

ZINC: Zain Innovation Campus
IT: Information Technology
BIG: Business Incubation Growth
